# Advanced microfluidic techniques for the preparation of solid lipid nanoparticles: Innovations and biomedical applications

**DOI:** 10.1016/j.ijpx.2025.100399

**Published:** 2025-09-15

**Authors:** Mohammad Javad Javid-Naderi, Seyed Ali Mousavi Shaegh

**Affiliations:** a Laboratory of Microfluidics and Medical Microsystems, Basic Sciences Research Institute, Mashhad University of Medical Sciences, Mashhad, Iran; b Orthopedic Research Center, Ghaem Hospital, School of Medicine, Mashhad University of Medical Sciences, Mashhad, Iran; c Clinical Research Unit, Ghaem Hospital, School of Medicine, Mashhad University of Medical Sciences, Mashhad, Iran

**Keywords:** Solid lipid nanoparticles, Microfluidic, Artificial intelligence, Drug delivery systems, Cosmeceutical applications, Industrial scale-up

## Abstract

Solid lipid nanoparticles (SLNs) represent a promising category of nanocarriers used in medicine and cosmetics, offering enhanced drug protection, controlled release, and targeted delivery for both hydrophilic and lipophilic compounds. Conventional preparation methods, such as high-pressure homogenization and solvent emulsification-evaporation, face several challenges, including increased polydispersity, scaling limitations, and the presence of hazardous residual solvents. Microfluidic technology has emerged as a novel approach for preparing SLNs, addressing issues such as variable particle sizes and residual solvents by facilitating enhanced control over particle dimensions, morphology, and encapsulation efficiency. Microfluidics enables rapid and uniform mixing through micro-scale fluid dynamics, resulting in the production of homogeneous nanoparticles with adjustable characteristics. The review examines key parameters in microfluidic SLN preparation and categorizes various microfluidic chip designs and mixing techniques in detail, illustrating their unique advantages in controlling nanoparticle properties. Furthermore, this article provides a comprehensive overview of microfluidic SLN preparation, emphasizing its advantages over conventional methods, and explores the transformative potential of SLNs for advancing drug delivery systems, cosmetic formulations, and diagnostics. The integration of artificial intelligence (AI) and machine learning to optimize synthesis conditions and enhance reproducibility and scalability for industrial translation are also discussed.

## Highlights

• Solid lipid nanoparticles (SLNs) prepared using microfluidic and conventional techniques were discussed and compared.
• Microfluidic mixer architectures for the preparation of SLNs were reviewed.
• Microfluidically prepared SLNs exhibit enhanced drug encapsulation, stability, and efficacy.
• The integration of AI/ML is a key future direction for scalable SLN manufacturing.
• Considerations for industrial-scale production of SLNs using microfluidics were provided.

## Graphical abstract

**Figure:**
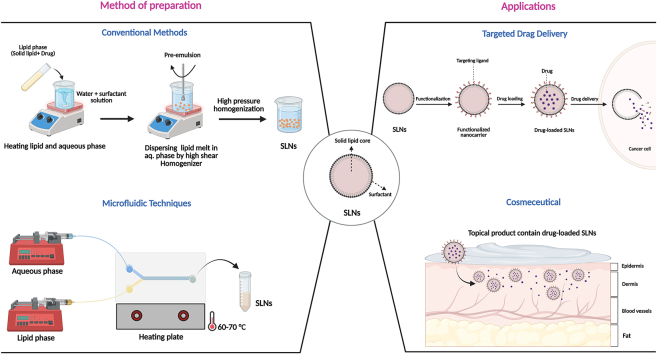

## 1 Introduction

Solid lipid nanoparticles (SLNs) are a type of drug delivery system that can carry both hydrophilic and lipophilic active pharmaceutical ingredients (APIs). These nanocarriers have a solid lipid core that is stabilized by surfactants or emulsifiers. They have many structural advantages, are very biocompatible, and can release drugs in a controlled way. The core of an SLNs is a lipid matrix that stays solid at both room temperature and body temperature. Physiologically compatible lipids like triglycerides, fatty acids, steroids, or waxes are usually used to make this matrix. Surfactants like phospholipids, lecithin, bile salts, polysorbates, or poloxamers are used to stabilize the lipid core and keep the particles from accumulation together in the aqueous suspension (Mishra et al., 2018; Duan et al., 2020; Arghidash et al., 2024). SLNs are spherical particles that range in size from 50 to 500 nm in diameter. This size makes it easier for them to penetrate the skin, deliver drugs to specific areas, and interact with biological membranes (A Review on Novel Delivery System: Solid Lipid Nanoparticles, 2019; Dobreva et al., 2020; Paliwal et al., 2020). SLNs have several important advantages over regular nanocarriers, such as better drug protection, longer circulation times, and the possibility of targeted delivery (N Kendre et al., 2024). The lipid matrix is solid, which makes it a stable place that keeps drugs that are sensitive to the environment (like oxidation, UV light, and enzymes) from breaking down and leaking out too soon. This matrix also makes it easier to control and keep drug release going, which can mean fewer doses are needed. Also, SLNs show high encapsulation efficiency for a wide range of drugs, especially those that don't dissolve well in water. This makes them more useful for treating a wider range of conditions. SLNs are safe to use in many ways because they are made from biodegradable lipids that are generally recognized as safe (GRAS) (Viegas et al., 2023; Rehman et al., 2024). These traits make SLNs very promising carriers for bioactive compounds in cosmetics, pharmaceuticals, and nutraceuticals (Arabestani et al., 2024; Satapathy et al., 2021). Even though SLNs have many benefits, they are not used as much as they could be because of problems with traditional preparation methods like high-pressure homogenization, solvent emulsification-evaporation, and ultrasonication (Subroto et al., 2023). For example, high-pressure homogenization can create a polydisperse particle population because the shear forces aren't always the same. Solvent-based methods, on the other hand, can leave toxic solvents in the final product. Microemulsion techniques are scalable; nonetheless, they frequently encounter issues with repeatability and lipid crystallization during storage that may lead to the release of medicines and a reduction in encapsulation efficiency. A significant drawback of these conventional procedures is their inadequate control over the size and morphology of the particles. These are critical parameters that influence the drug's efficacy and its distribution throughout the body. All these challenges highlight the need to develop improved platforms for nanocarrier preparation that enhance scalability, uniformity, and standardization of methods (Daneshmand et al., 2018; Musielak et al., 2022). Microfluidic technology has emerged as a transformative and cutting-edge solution for the preparation of various nanocarriers including SLNs, addressing the limitations of traditional methods (Khoshnood et al., 2025; Dangkoub et al., 2024). By enabling precise manipulation of fluid dynamics and mixing parameters at the microscale, microfluidic platforms facilitate the production of highly monodisperse SLNs with uniform size distribution, tunable sizes, and enhanced encapsulation efficiencies for hydrophobic drugs (Bezelya et al., 2023; Osouli-Bostanabad et al., 2022). The integration of advanced features, like herringbone micromixers and 3D hydrodynamic flow focusing, boosts mixing efficiency, speeds up solvent blending, and ensures even lipid formation. It also tackles problems like clogging and increases output. These systems allow fine-tuning of key factors like total flow rate (TFR) and flow rate ratio (FRR), making it possible to tailor SLN properties to meet specific needs in biology and cosmetics. Microfluidics stands out from conventional batch-based methods by offering continuous and scalable production. This leads to greater reliability and consistency while also using less solvent, energy, and waste. Additionally, high-throughput capabilities of microfluidic platforms make them ideal for industrial translation for mass production of nanocarriers. With these advantages, microfluidic technology is increasingly recognized as the gold standard for environmentally friendly, cost-effective, and scalable SLNs production tailored for diverse applications (Mehraji and DeVoe, 2024; Carvalho et al., 2022).

This review aims to provide a comprehensive overview of advances in microfluidic preparation of SLNs, focusing on their biomedical applications. It highlights the capabilities of microfluidic platforms for producing SLNs with controlled size, morphology, and drug encapsulation efficiency. This review also addresses the challenges and future perspectives of microfluidic SLN preparation, providing insights into the potential of this technology for advancing nanomedicine and cosmeceutical development.

## 2 Preparation methods of SLNs

### 2.1 Conventional methods

**Fig. 1:**
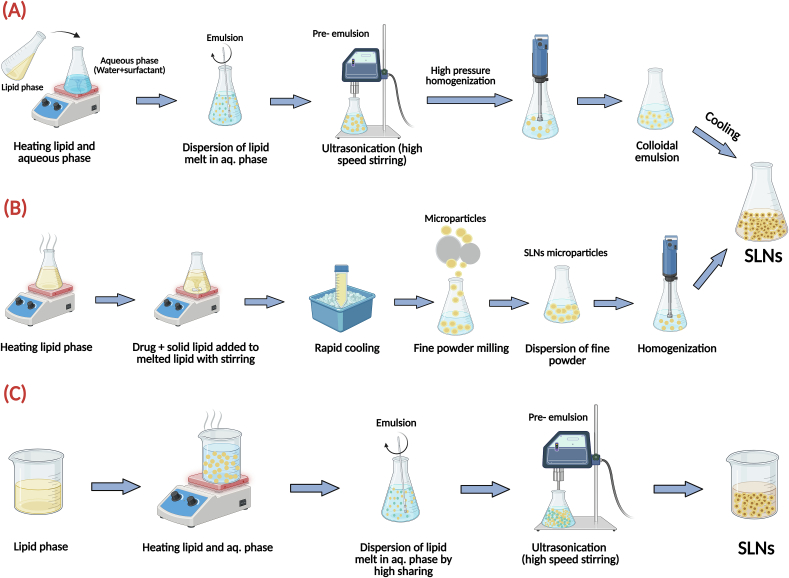
High-energy mechanical methods for the preparation of SLNs. (A) The hot homogenization process uses high pressure on a heated emulsion. (B) Cold homogenization protects thermolabile drugs by processing a pre-milled solid lipid dispersion at low temperatures. (C) Ultrasonication employs high-frequency sound waves to fragment lipid droplets into nanoparticles.

**Fig. 2:**
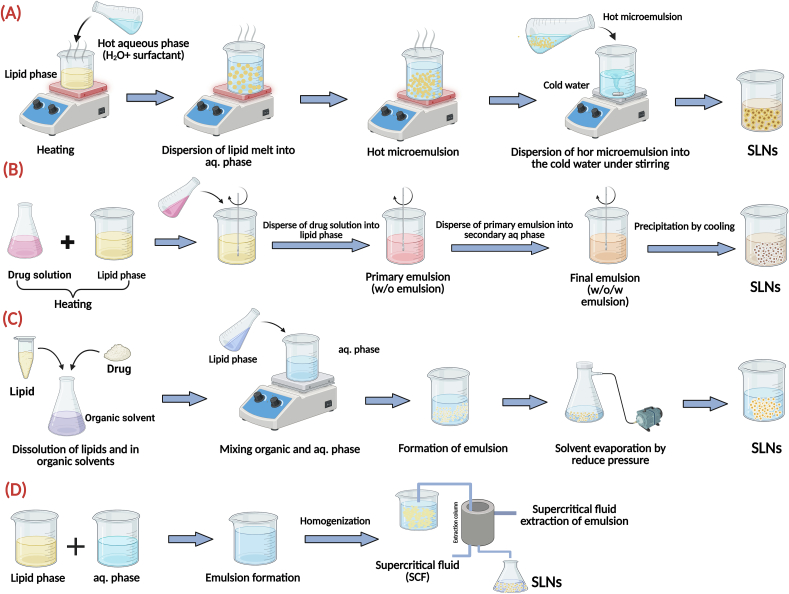
Emulsion and solvent-based methods for the preparation of SLNs. (A) The microemulsion technique relies on diluting a stable microemulsion to precipitate SLNs. (B) The double emulsion method is used to encapsulate hydrophilic drugs. (C) Solvent emulsification-evaporation involves removing a volatile organic solvent to form nanoparticles. (D) The supercritical fluid technique uses an environmentally friendly supercritical fluid as a solvent.

Conventional methods for preparation of SLNs present a diverse array of techniques, each possessing inherent advantages and limitations that influence their suitability for specific applications, and broadly categorized into high-energy mechanical approaches **(**Fig. 1**)** and those based on emulsion and solvent manipulation **(**Fig. 2**)**. High-energy methods like high-pressure homogenization (both hot and cold) and ultrasonication utilize intense physical forces to reduce particle size. In contrast, other techniques rely on the principles of spontaneous emulsification or solvent removal.

Hot high-pressure homogenization (HPH) is the most popular, commercially viable method for the preparation of SLNs. In this case, the hot pre-emulsion, prepared by the dispersion of molten drug-lipid in the hot aqueous surfactant phase, is forced at high pressures of 500–1500 bar through the narrow orifice (Fig. 1(A)). Owing to the high temperature, the viscosity of the lipids is decreased, facilitating the formation of smaller particles upon further cooling and recrystallization. Though strong and reproducible, the method is not suitable for thermolabile drugs since the latter can undergo heat degradation. This method is also not efficient for hydrophilic drugs, since the latter partition in the outer water phase, resulting in low encapsulation efficiency accompanied by high burst release (Pareek et al., 2025; Nguyen and Duong, 2022). Cold HPH was method was developed to overcome these limitations. This is one of the ways the drug is dissolved in a melt of lipids, cryogenically frozen, and milled into microparticles (typically 50–100 μm). These microspheres are suspended in a cold surfactant solution and homogenized at lower temperatures (e.g., 0–4 °C) (Fig. 1(B)). Its most significant advantage is the ability of this technique to protect temperature-sensitive drugs as well as to enhance hydrophilic compound loading by decreasing the compound loss to the aqueous phase. However, the drawback is the more laborious procedure, which leads to the formation of bigger, more polydisperse nanoparticles compared to the hot HPH method (Akanda et al., 2023). Ultrasonication of SLNs preparation is achieved through the application of sound waves at a high frequency to create cavitation inside the lipid dispersion (Fig. 1(C)). Vacuum bubbles are formed by sound waves that capture energy but implode, causing significant transmembrane pressure gradients that disrupt multilamellar vesicles, dividing larger droplets of the lipids into smaller droplets as well as creating nanoparticles. It has always been used together with high-speed stirring (high-shear homogenization) for increased efficiency and smoothness. Favored for its ease of application and economy in the laboratory, however, its wider application has always been frustrated by severe limitations. These are poor control over the size of the resultant particles, leading to wide polydispersity; danger of metal contamination from sonication probes; possibility of thermal degradation of labile ingredients; as well as poor long-term physical stability of the formulation (Biswas et al., 2024).

The microemulsion technique is a low-energy and straightforward method for preparing SLNs. The method starts with the formation of a thermodynamically stable o/w micro-emulsion, which results from gentle mixing of the melted lipid-drug phase with an aqueous phase containing co-surfactants along with surfactants at the same temperature. The thus-formed microemulsion droplets, on fast dispersion of hot microemulsion into a tremendous excess volume of cold aqueous phase, turn solid on sudden change of temperature, forcing precipitation as well as recrystallization of the lipid into nanoparticles, upon which the final size of the droplets rests significantly on the rate of such dispersion step (Fig. 2 **(A)**) (Yasir et al., 2018; Masiiwa and Gadaga, 2018).

Double emulsion technique, commonly based on water-in-oil-in-water (W/O/W) emulsion method, is a widely employed approach for the preparation of SLNs, particularly for the encapsulation of hydrophilic drugs (Fig. 2 **(B)**). This method involves two sequential emulsification steps. Initially, a primary emulsion is formed by dissolving a drug in an aqueous phase, which is then emulsified with a lipid phase. Subsequently, this primary emulsion is further emulsified with an external aqueous phase to produce the final double emulsion. The lipid phase typically consists of biocompatible lipids such as stearic acid, glyceryl monostearate, or cetyl palmitate, while surfactants such as Tween 80 and Span 80 are commonly used to stabilize both primary and secondary emulsions. This technique offers significant flexibility in the selection of lipids and surfactants, enabling the optimization of critical parameters such as particle size, stability, and drug encapsulation efficiency. The double emulsion method is thus a versatile and effective strategy for designing SLNs tailored to specific drug delivery applications (Sastri et al., 2020; Stefanov et al., 2023). In solvent-based methods, such as emulsification-diffusion and evaporation, the lipid and drug are first dissolved in a volatile organic solvent (Fig. 2 **(C)**). This organic phase is then emulsified within an aqueous solution, and the solvent is subsequently removed via evaporation or diffusion. Although these methods are valued for their operational simplicity and scalability, they present an inherent risk of residual solvent contamination in the final product (Mohammed et al., 2023). The supercritical fluid-based approaches utilize supercritical fluids, such as carbon dioxide, as solvents to precipitate lipids and drugs into nanoparticles following rapid expansion (Fig. 2 **(D)**). This approach is environmentally safe and suited for heat-sensitive compounds but requires specialized, high-cost equipment. Each of these approaches affects the resultant physicochemical properties of SLNs, hence influencing their appropriateness for particular drug delivery applications (Navaneetha Krishnan et al., 2024). Beyond any particular method, numerous common problems plague conventional SLNs preparation and hinder industrial adoption. Variability across batches is a major issue. Even modest processing parameter or raw material changes might severely impact product quality. Scalability is another major difficulty. Industrial manufacturing of a laboratory-scale formulation requires substantial equipment design and process parameter adjustment. For repeatable drug administration, particle size, shape, and crystallinity must be maintained during this transition. Also, commercial viability requires long-term colloidal stability of the formulation throughout storage and transport, with major efforts to prevent particle aggregation and drug expulsion. Finally, clinical and commercial formulations must follow regulatory criteria, especially those regarding residual organic solvent limitations and biocompatible excipients (Naseri et al., 2015). Addressing the above-mentioned limitations is pivotal for more implementation of SLNs in pharmaceutical and cosmeceutical applications. Consequently, ongoing research efforts are increasingly focused on exploring novel approaches, such as microfluidic technologies, which offer enhanced control over particle formation and potentially overcome the limitations of conventional methods (Khairnar et al., 2022).

Table 1 provides a comprehensive list of conventional preparation methods for SLNs, including their benefits, drawbacks, obstacles, and prevalent industrial concerns.

**Table 1:** Features of the conventional methods to prepare SLNs.

Method	Advantages	Disadvantages	Challenges	Common industrial problems	Ref.
Hot Homogenization	Scalable process, relatively simple process, narrow size distribution, high encapsulation efficiency, applicable to lipophilic drugs, enhanced drug solubility and stability	Potential degradation of thermolabile drugs, high energy input, risk of supercooled melts	Optimizing process parameters (temperature, pressure, number of cycles), avoiding particle aggregation, controlling crystallization process	Maintaining thermal stability during processing, temperature control in large batches, complexity in scale-up and maintaining product quality, equipment maintenance and cleaning challenges	(Jana et al., 2024)
Cold Homogenization	Suitable for heat-sensitive compounds, avoids drug degradation	Broader size distribution, lower encapsulation efficiency, requires pre-emulsion step	Optimizing milling and homogenization conditions, ensuring complete drug solubilization in lipid melt, achieving desired particle size, maintaining low temperature throughout process	Difficulty in reproducibility, challenges in scaling up the process, energy-intensive cooling, potential agglomeration during storage, time efficiency in production	(Jana et al., 2024)
Ultrasonication	Cost-effective and easy to use, no organic solvents required, suitable for small-scale production.	Potential for particle aggregation, high energy input may degrade sensitive drugs, non-uniform particle size distribution, risk of metal contamination from probes.	Limited control over particle size, Risk of contamination from probe sonicators, requires extensive optimization to prevent particle aggregation	Poor scalability for large batches, aggregation during storage	(Kumar et al., 2018; Sreeharsha et al., 2024)
Microemulsion	Simple and reproducible process, suitable for lab-scale production, high encapsulation efficiency and stability, no need for high temperatures or high-pressure equipment.	Requires high amounts of surfactants and co-surfactants, residual surfactants may cause toxicity, limited scalability, sensitive to environmental conditions like temperature and pH, limited to certain types of lipids, requires dilution, leading to instability	Optimizing surfactant-to-lipid ratios, ensuring long-term stability, controlling particle size is difficult, high costs associated with surfactants and co-surfactants, removing excess water from final product, ensuring complete solvent elimination	Limited industrial scalability, difficult to achieve consistent quality during large-scale manufacturing, challenges in large-scale production, Surfactant removal in final product, Residual solvent toxicity, high production costs for solvent recovery.	(Chirio et al., 2019; Kotmakçı et al., 2017)
Double Emulsion	Controlled release, high encapsulation efficiency, biocompatibility	Complexity, particle size variability, limited drug loading capacity	Optimization of formulation parameters, maintaining particle size consistency	Scale-up, high energy requirements, batch-to-batch variability	(Rajpoot, 2019)
Solvent Emulsification-Evaporation	Mild processing conditions, ability to encapsulate both hydrophilic and hydrophobic drugs, relatively simple setup, produces compact nanoparticles with high drug entrapment, suitable for thermolabile compounds, good control over particle size, No thermal stress during preparation	Use of organic solvents, which may be toxic, potential for drug loss during the solvent evaporation step, difficulty in controlling particle size, time-consuming process	Optimization of factors like lipid content, surfactant, and co-surfactant, ensuring complete solvent removal, maintaining stability during evaporation	Difficulty in scaling up the process, concerns about residual solvent levels, high production costs for solvent recovery	(Duong et al., 2020; Pooja et al., 2016)
Supercritical Fluid Technology	Eco-friendly and solvent-free process, Single-step process, precise control over particle size, suitable for heat-sensitive drugs.	High equipment and operational costs, requires specialized expertise, limited to specific lipids soluble in SCF	High cost of supercritical fluid equipment, low throughput for large-scale production, requires optimization of pressure and temperature parameters	Expensive setup and maintenance costs, scalability issues for large-scale production, specialized expertise required	(Trucillo and Campardelli, 2019)

### 2.2 Microfluidic approaches

#### 2.2.1 Fundamentals of microfluidic synthesis

Microfluidic synthesis utilizes the unique characteristics of fluid manipulation at the microscale to provide exact control over chemical processes and material development. Microfluidic systems have numerous advantages over conventional bulk synthesis methods by restricting reactions to microchannels, which generally measure tens to hundreds of micrometers. These benefits arise from the unique physical environment established within microfluidic devices. Microfluidics is a technology that accurately processes and manipulates fluids in volumes between 10^−9^ to 10^−18^ L in microchannels with dimensions generally from 10 to 1000 μm. This miniaturized fluidic environment exhibits unique physical characteristics compared to the macroscopic world due to significantly enhanced surface effects (Shepherd et al., 2021; Nguyen et al., 2019; Liu et al., 2021b). Microfluidics is regulated by numerous fundamental principles that define its characteristics and applications. A key component is laminar flow, which predominates in microchannels due to the low Reynolds numbers normally observed at this scale. This leads to fluid layers running in parallel with minimal turbulence, resulting in mixing mostly reliant on molecular diffusion at the interfaces of neighboring fluid streams (Fan et al., 2019). In this way, controlled mixing of streams is achievable. Another critical feature is the enhanced surface effects arising from high surface-to-volume ratio in microchannels, which significantly impacts fluid behavior, including wetting phenomena and contact angles factors essential for the optimal design and performance of microfluidic devices (Xie et al., 2022). Additionally, short diffusion distances inherent to microchannels enable rapid mass and heat transfer, facilitating efficient mixing and accelerated reaction kinetics. Owing to these facts, microfluidics provides a precise control over mixing rates of the streams, reaction times and temperature to create platforms for tailored synthesis and chemical processes, such as production of nanocarriers. Microfluidic systems are mainly divided into two categories: continuous-flow microfluidics, which employs miscible fluids for steady-state operations, and segmented-flow microfluidics, which utilizes immiscible fluids to generate droplets or discrete segments, thereby establishing isolated reaction environments essential for applications necessitating compartmentalization. Collectively, these concepts and categories support the adaptability and effectiveness of microfluidic technology across various scientific and industrial applications (Zhang et al., 2023; Shen et al., 2024).

#### 2.2.2 Preparation of SLNs using microfluidic techniques

Conventional bulk manufacturing techniques for SLNs exhibit numerous disadvantages, such as inadequate repeatability, elevated polydispersity, variability across batches, and challenges in scaling for commercial production (Bukke et al., 2024). Microfluidic technology provides precise regulation of fluid dynamics within microchannels to overcome these drawbacks. A significant advantage of microfluidic platforms is their superior control over particle size and dispersion. By precisely regulating flow rates and channel geometry, very uniform nanoparticles with low polydispersity can be produced for reliable drug delivery. This control ensures high batch-to-batch reproducibility, essential for clinical translation and regulatory approval. Microfluidics promotes environmental and economic sustainability by reducing solvent and energy use. The technology enables the deliberate design of SLNs with tailored drug release profiles through precise control of nanoparticle formation (Garg et al., 2016; Fabozzi et al., 2023).

**Fig. 3:**
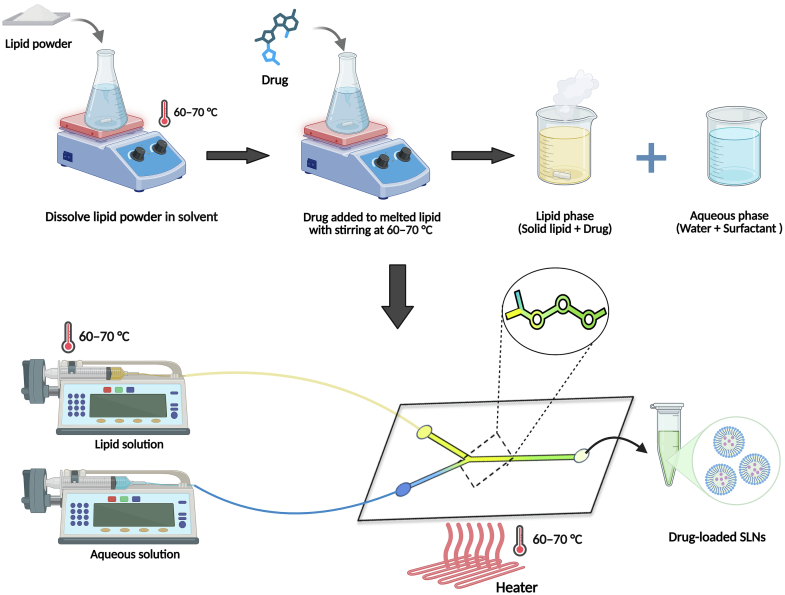
Set-up for the preparation of SLNs via microfluidic chip. The microfluidic mixing chip is heated to maintain temperature during SLNs production.

The fundamental preparation method of drug-loaded SLNs by microfluidic techniques is a multi-step, highly controlled procedure triggered by the formation of two individual solutions: an aqueous solution and an organic solution. The organic solution is prepared by the dissolution of the chosen solid lipid (e.g., cetyl palmitate or tristearin) together with the lipophilic drug in a water-miscible solvent, preferably ethanol. The aqueous solution, by contrast, is generated by the dissolution of a stabilizing surfactant (e.g., PEGylated lipid or Poloxamer 188) within a buffer solution. The two solutions thus prepared are forced into the microfluidic mixer chip by high-precision syringe pumps at controlled rates. In the chip, within a laminar regime of flow, the streams mix rapidly but periodically, often with the aid of special geometry mixers. The resultant rapid mix leads to the diffusion of the ethanol to the aqueous stream to yield a state of supersaturation, on account of which the co-precipitation of the lipid with the drug takes place with the formation of the SLNs by self-assembly. The final nanoparticle characteristics are precisely tuned by regulating the operating variables like the Total Flow Rate (TFR) and the Flow Rate Ratio (FRR). The crude dispersion collected from the chip outlet is laden with residual solvent and unencapsulated drug, necessitating a critical purification step (Prakash et al., 2022; Jaradat et al., 2021). The preparation of drug-loaded SLNs is performed using a continuous microfluidic technique, as displayed in Fig. 3. The method involves preparing a hot lipid phase (60–70 °C) containing the dissolved drug, as well as a separate aqueous phase containing the surfactant. These two solutions are then pumped into a temperature-controlled microfluidic chip. Inside the device, rapid and controlled mixing of the two streams results in the formation of nanoparticles. The resulting suspension of SLNs containing encapsulated drug molecules is then collected from the chip outlet for further characterization and use. Maintaining a constant temperature throughout the chip is crucial to prevent premature solidification of the lipid and to ensure reproducible nanoparticle properties.

#### 2.2.3 Key parameters in microfluidic nanocarrier preparation

**Fig. 4:**
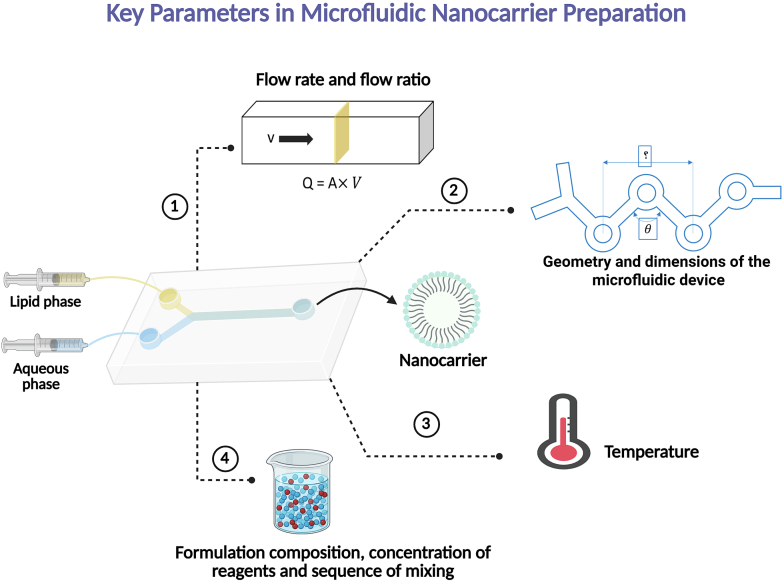
Key parameters influencing nanocarrier preparation in microfluidic systems. These parameters include flow rate and ratio, device geometry, temperature, formulation composition, concentration of reagents, and sequence of mixing, all of which collectively determine the final properties of the formed nanocarriers.

The key parameters in the microfluidic preparation of nanocarriers like SLNs are interdependent factors that must be optimized to control the final properties of the nanoparticles. Key parameters that influence the characteristics of microfluidically prepared nanocarriers include flow rates, device geometry, formulation composition, and temperature. A fundamental aspect of microfluidic synthesis is the precise control over fluid dynamics within the device. In typical experimental setups, the volumetric flow rate (Q) of each fluid stream is the primary parameter controlled externally, for instance by a syringe pump. This externally set flow rate, in conjunction with the fixed cross-sectional area (A) of the microchannel, dictates the average velocity (V) of the fluid according to the relationship (Q = A × V) (Mudugamuwa et al., 2024; Shrirao et al., 2018). This control is leveraged through two key experimental parameters: the total flow rate (TFR), which is the sum of the individual stream flow rates, and the flow rate ratio (FRR) between the fluid streams. These parameters are particularly significant as they determine the mixing efficiency, the residence time within the device, and ultimately, the size of the formed nanoparticles. While higher flow rates can enhance mixing in specific designs, they also reduce residence time, potentially affecting nanoparticle formation. The FRR is especially critical in nanoprecipitation processes, as it governs the level of supersaturation between the solvent and antisolvent phases, directly influencing the nucleation and growth of nanoparticles (Osouli-Bostanabad et al., 2022). Additionally, the geometry and dimensions of the microfluidic device play a crucial role on mixing rate. Features such as shape, length, and incorporation of specific mixing structures, e.g., baffles or herringbone patterns, control fluid dynamics and enhance mixing efficiency. These geometries promote mixing through mechanisms such as hydrodynamic focusing, chaotic advection, or increased interfacial contact between fluids. Mixing time and efficiency are equally critical, as rapid and effective mixing ensures uniform nanoparticle formation with controlled size and low polydispersity. Mixing time is influenced by both device geometry and flow rates, with efficient mixing facilitating homogeneous nucleation and growth processes (Liu et al., 2022). Moreover, temperature and pressure within the microfluidic system can profoundly influence nanoparticle synthesis. Temperature influences the solubility of precursors and the kinetics of nanoparticle synthesis, whereas pressure predominantly regulates the flow rate in microfluidic systems. Higher pressure increases flow rates, which research suggests leads to smaller nanocarriers due to faster mixing, reducing aggregation time (Maeki et al., 2017). Finally, formulation composition, the concentration of reagents, and the sequence of mixing are crucial factors. The initial concentrations of precursors, including lipids, polymers, or drugs, in their respective solvent streams significantly influence the physicochemical properties of the nanoparticles. Similarly, the sequence in which reagents are introduced and mixed can influence self-assembly processes and the encapsulation efficiency of therapeutic agents (Vogelaar et al., 2023). The precise control over these parameters afforded by microfluidics shifts the nanoparticle formation process from being thermodynamically driven, as is often the case in bulk methods, to being kinetically controlled. This means that the rate of mixing and solvent exchange, rather than just the final equilibrium state, dictates the characteristics of the nanoparticles. This kinetic control allows for the precise manipulation of nucleation and growth phases, enabling the production of highly uniform and size-controlled nanoparticles. This is a significant advantage over conventional methods, where non-uniform dilution of ethanol is a primary reason for batch-to-batch variation (Maeki et al., 2022). Fig. 4 highlights the key parameters influencing microfluidic nanocarrier preparation. Together, these parameters are interdependent and must be optimized to achieve precise control over the properties of the resulting nanocarriers.

### 2.3 Microfluidic device engineering

#### 2.3.1 Materials and fabrication methods of microfluidic chips

Materials used in microfluidic chip fabrication play a pivotal role in determining the characteristics of the synthesized nanomaterials, as they amplify surface properties at the microscale. The final properties of the SLNs can be influenced by changes in drug concentration and flow dynamics resulting from the interaction between the channel surface and the fluids, particularly during continuous operations (Gimondi et al., 2023). A wide range of materials can be processed into miniaturized chips with microscale channels and chambers, and their selection depends on factors such as durability, ease of fabrication, transparency, biocompatibility, chemical compatibility, resistance to temperature and pressure, and potential for surface functionalization. The most popular materials, which provide a trade-off between cost, ease of fabrication, and chemical performance, are polydimethylsiloxane (PDMS), glass, and thermoplastics (Zhao et al., 2020; Niculescu et al., 2021; Ren et al., 2013). PDMS a flexible, low-cost, and biocompatible elastomer, is widely used as a material for microfluidic chips. However, its properties pose serious challenges for drug development (Alghannam et al., 2025). PDMS is absorbent and hydrophobic. This property is undesirable for drug delivery applications because it leads to nonspecific adsorption of various molecules (Li et al., 2009). Hydrophobic small-molecule drugs, proteins, and lipids can easily diffuse into the channel or stick to the channel walls. Such adsorption leads to a reduction in the effective drug concentration and consequently to a significant loss of therapeutic load with unpredictable dosing. For protein-based drugs, this can also lead to protein denaturation, thereby compromising their biological activity. This material behavior directly affects drug stability and can lead to clogging of the channel, which limits long-term and continuous performance (Auner et al., 2019; Zhang and Chiao, 2015). One notable disadvantage of PDMS is its poor compatibility with organic solvents, such as ethanol and chloroform, which are commonly used to prepare lipid-based nanodrugs, including SLNs. PDMS deforms and swells when exposed to such solvents, disrupting both the flow dynamics and the geometry of the channels (Dangla et al., 2010). This lack of chemical resistance complicates achieving stable process conditions, resulting in variations between batches of nanoparticles. Deformation of PDMS when equipment is operated at high pressure, typically required for adequate mixing, disrupts the reproducible fluid flow necessary for the controlled assembly of nanoparticles. This results in poor control of SLN size and size distribution (monodispersity), which are quality characteristics relevant to drug delivery systems (Bi et al., 2025). Glass has long been regarded as the gold standard for microfluidic applications involving chemical inertness and exact requirements, hence why it is suited for pharmaceutical production. Glass has chemical inertness, as well as a hydrophilic surface, which results in minimal adsorption of the hydrophobic molecules, the lipids, as well as proteins, thus ensuring enhanced stability and pharmaceutical recovery of therapeutic agents. This is a substantial advantage over PDMS, as it prevents drug loss and maintains the integrity of sensitive biomolecules. The minimal non-specific binding ensures accurate and reproducible dosing, which is essential for drug delivery (Aralekallu et al., 2023). Glass exhibits outstanding chemical resistance to numerous organic solvents, increased temperatures, and pressures. It does not swell nor deteriorate, providing rugged, highly reproducible continuous processing regardless of solvent system choice. The stability guarantees the reproducibility of process parameters necessary for the reproducible production of nanocarriers. Stable and rigid glass channels guarantee accurate control of nanoparticle properties (Elvira et al., 2022; Scott and Ali, 2021).

Thermoplastics such as polymethyl methacrylate (PMMA), polycarbonate, polylactic acid (PLA), polyvinyl chloride (PVC), and cyclic olefin copolymer (COC) are commonly used to develop microfluidic devices. Thermoplastics can be engineered easily using high precision micromachining methods such as milling or laser ablation methods to create desired channel geometries that significantly enhance mixing efficiency and enable the production of small, monodisperse nanoparticles (Banejad et al., 2020; Shaegh et al., 2018). Thermoplastics offer a balance between fabrication ease (compared to glass) and performance, enabling the consistent production of high-quality nanoparticles (Shakeri et al., 2022). From industrial point of view, it is worthy to mention that gold standard material for pharma and biotech industry is stainless steel that can be formed and machined according to requirements and present high resistant to solvents and chemical used during drug production and also during washing and cleaning protocols (Gencturk et al., 2017). There is a wide range of fabrication methods utilized to create microfluidic chips, each having various benefits as well as limitations. Photolithography is a primary technique, depositing complex, high-precision designs onto photopatternable substrates, such as silicon or glass. It is highly effective for complex designs, but its application has limitations due to high costs and the need for specialized equipment and cleanroom facility space. Building on this, soft lithography offers a more accessible and cost-effective alternative. This method typically utilizes a silicon master mold, often fabricated through photolithography, to cast elastomeric materials such as PDMS (Nguyen et al., 2022). For mass production and complex geometries, mechanical and additive manufacturing methods are increasingly utilized. Hot embossing and injection molding are well-suited for shaping thermoplastic materials, such as PMMA and COC, by applying heat and pressure with a mold. These processes are highly effective for large-scale manufacturing (Konstantinou et al., 2016; Lee et al., 2018). Similarly, micromilling offers a rapid mechanical approach for prototyping plastic devices (Allen et al., 2024). In contrast, 3D printing, or additive manufacturing, permits complex three-dimensional geometries to be shaped, layer by layer, which standard methods cannot readily achieve. It has, to date, despite permitting rapid prototyping as well as an enormous design space, the disadvantage of inadequate z-resolution, as well as a limited set of optically transparent materials (Aldaghestani et al., 2025; Toosi et al., 2024). Other direct-writing methods, such as laser ablation, offer rapid material removal to create complex geometries in various substrates, providing a simplified alternative to conventional lithography (Mansour et al., 2023). Finally, etching is one of the primary channel-forming techniques used on glass and silicon. Wet etching, utilizing aggressive chemical etchants, permits rapid processing of large wafers but can create isotropic profiles, as well as cause problems stemming from the toxic compounds used. Dry etching, utilizing directional ion bombardment, produces well-defined, anisotropic channel profiles but slows processing and increases costs through the requirement of specialized equipment (Konstantinova et al., 2023).

#### 2.3.2 Integrated temperature control systems

Temperature regulation during mixing may be essential to keep the lipid in a molten state for uniform mixing (Trivino et al., 2019; Souto et al., 2019). Temperature control is a critical aspect of SLN preparation, particularly when using solid lipids that require melting for proper dissolution and homogeneous mixing with aqueous phase. The temperature during the preparation process can significantly influence the drug loading capacity and the subsequent drug release profiles of SLNs (Musielak et al., 2022). Various heating methods have been integrated into microfluidic systems to provide the necessary temperature control for SLNs preparation. External heating sources, such as heating stages or oil baths, can be used to heat the entire microfluidic device or the solutions prior to their introduction into the chip (Kulkarni and Goel, 2022). Previous studies have demonstrated the utility of these heating methods for the preparation of microfluidic SLNs. A recent study by Arduino et al. utilized a temperature controller integrated with a Kapton film heater that was attached to the organic phase reservoir and the microfluidic chip to maintain a constant temperature for the preparation of cetyl palmitate-based SLNs. This study employed a glass capillary microfluidic system to fabricate SLNs composed of cetyl palmitate and DSPE-PEG, dissolved in a 25 % ethanol solution, subsequently combined with an aqueous stabilizer solution. The main influencing parameters comprised flow rates, surfactant types (e.g., Pluronic F68, Tween 80), and most importantly, temperature, which was sustained above 60 °C for the entire process. The temperature control was achieved by surrounding the lipid-filled syringe with an electric wire linked to a pressure regulator and submerging the microfluidic chip in a 60 °C water bath. The heating function was crucial; it kept the lipids in a liquid state during nanoprecipitation, preventing premature solidification that could lead to larger or aggregated particles. The implementation of an enhanced microfluidic device that optimized fluid mixing enabled the fabrication of SLNs with an average diameter of approximately 121 nm and a significantly lower polydispersity index (0.11) compared to those produced by the bulk method. The results were highly promising; SLNs exhibited enhanced colloidal stability in physiologically relevant media, including PBS, cell culture medium, and human plasma, alongside elevated encapsulation efficiencies for drugs such as paclitaxel (PTX) and sorafenib (SFN) (Arduino et al., 2021b). Another study Kim et al. successfully developed stable and uniform curcumin-loaded SLNs (Cur-SLNs) using a microfluidic system specifically modified with an integrated temperature controller. This approach was designed to overcome the low reproducibility and poor encapsulation efficiency common to conventional bulk manufacturing methods. The process involved maintaining the system at approximately 60 °C using film heaters on the organic phase reservoir, sample cylinder, and microfluidic chip, which is crucial for solid lipids like cetyl palmitate (melting point ∼55 °C) to remain liquid for nanoparticle synthesis. This temperature-controlled process resulted in the formation of nanoparticles with a uniform size of approximately 100–150 nm, a low polydispersity index, and a high encapsulation efficiency of over 76 %, compared to ∼40 % for conventional bulk methods. The prepared Cur-SLNs demonstrated sustained drug release over approximately six days and maintained colloidal stability for two weeks (Kim et al., 2025). Based on the study by Sommonte et al., microfluidic-based production of lysozyme-loaded SLNs (LZ-SLNs) demonstrated notable advantages compared to conventional bench-top methods. The microfluidic process was optimized by adjusting the TFR and the FRR, culminating in an optimal nanoformulation produced at a TFR of 30 mL/min and an FRR of 1:5 (organic to aqueous phase). This technique required precise temperature control, with the lipid phase (cetyl palmitate in ethanol) heated by an infrared lamp and the microfluidic chip maintained at 60 °C to prevent lipid precipitation. The microfluidic technique yielded LZ-SLNs with a significantly lower size (158.05 ± 4.86 nm) and higher encapsulation efficiency (70.15 ± 1.65 %) when compared to particles produced conventionally (180.21 ± 7.46 nm and 53.58 ± 1.13 %, respectively). Furthermore, in vitro studies confirmed that the SLNs provided a sustained release of the enzyme over seven days, and the released lysozyme retained its full biological activity (Sommonte et al., 2023).

The development of integrated and automated temperature control with precise flow injection into a mixing chip, which overcomes the limitations of manual laboratory setups and ensures reproducibility in formulation development for lipids with high melting points, is critical for clinical and industrial translation. To this end, heating and temperature control systems have been integrated with the injection of solutions in commercially available apparatuses such as NanoAssemblr™ Ignite system and NanoSynthes INSIGHT**®** as state-of-the-art commercial platforms that have effectively addressed the challenge of temperature control in SLNs preparation (NxGen, 2025; NanoSynthes, 2025).

### 2.4 Post-synthesis processing and microscopic characterization

Following synthesis, the obtained crude SLNs dispersion requires downstream processing for purity, stability, and clinical suitability. The processing begins with purification, the step necessary for the removal of excess organic solvents, non-encapsulated drugs, and excess surfactants. Though small-scale lab methods of dialysis are flexible, Tangential Flow Filtration (TFF) remains the favored standard, offering scalable, high-throughput purification and preventing the tendency for the formation of the irreversible aggregation characteristically induced by high-force methods like centrifugal ultrafiltration. The resulting purified aqueous dispersion is, thereafter, lyophilized (freeze-dried) to obtain the stable dry powder for long-term storage. The step is critically dependent on the addition of cryoprotectants, such as trehalose, which form a protective glassy matrix that prevents particle fusion and preserves the SLNs' structural integrity during the stresses of freezing and drying (Elbrink et al., 2023; Wu et al., 2025).

**Fig. 5:**
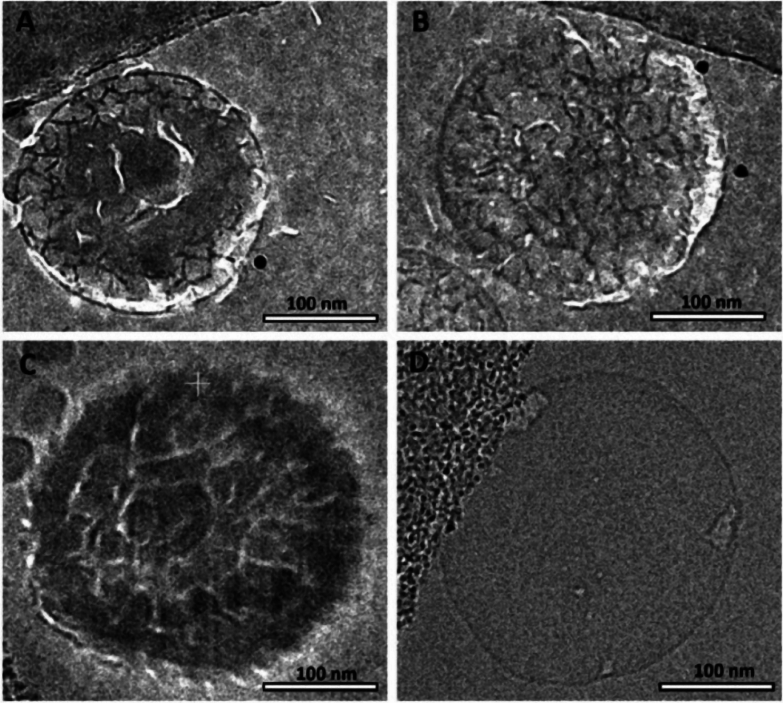
Cryo-EM micrographs of the turtle-like SLNs. (A) Empty of SLNs prepared by bench-top method. (B) LZ SLNs prepared by bench-top method. (C) Empty of SLNs prepared by microfluid technique. (D) LZ SLNs prepared by microfluidic technique (Sommonte et al., 2023) (The figure is adapted with permission from Elsevier).

For microscopic characterization, it is necessary to take a multi-modal approach since it is not possible for a single technique to achieve a full image of the SLNs system. Dynamic Light Scattering (DLS) gives ensemble information on mean particle size and PDI, but has no morphological information. Transmission Electron Microscopy (TEM) is useful for the direct measurement of particle size, distribution, and general morphology, substantiating spherical shapes, and identifying features such as core-shell structures. Scanning Electron Microscopy (SEM) is employed primarily to evaluate the final, solid-state (lyophilized) product's morphology, critically reporting the success of the lyophilization process by reporting whether nanoparticles are discrete or merged to become an aggregated mass. Atomic Force Microscopy (AFM) yields quantitative 3D topography measures, providing information on particle height as well as surface roughness. The “gold standard” for imaging SLNs in the native, hydrated state and uncovering their complex internal nanostructure is Cryo-Transmission Electron Microscopy (Cryo-TEM). By freeze-inducing samples rapidly into vitreous ice, Cryo-TEM circumvents artifacts of preparation, enabling measurements with high precision of size, shape, and important details as small as the lamellarity of the shell lipid, distinct phases of the lipids within the core, and even the encapsulated cargo (e.g., strands of siRNA or mRNA) (Queiroz and Muehlmann, 2024; Anderluzzi et al., 2019; Jamous et al., 2023). The choice of lipid composition and surfactant concentration profoundly impacts SLN morphology and internal structure. Cryo-TEM studies of lysozyme-loaded SLNs prepared via microfluidics highlighted a peculiar “turtle-like” structure on their surface. This feature represents another distinct morphological outcome influenced by the specific formulation and cargo **(**Fig. 5**)**.

## 3 Microfluidic mixer design and formulation case studies

**Fig. 6:**
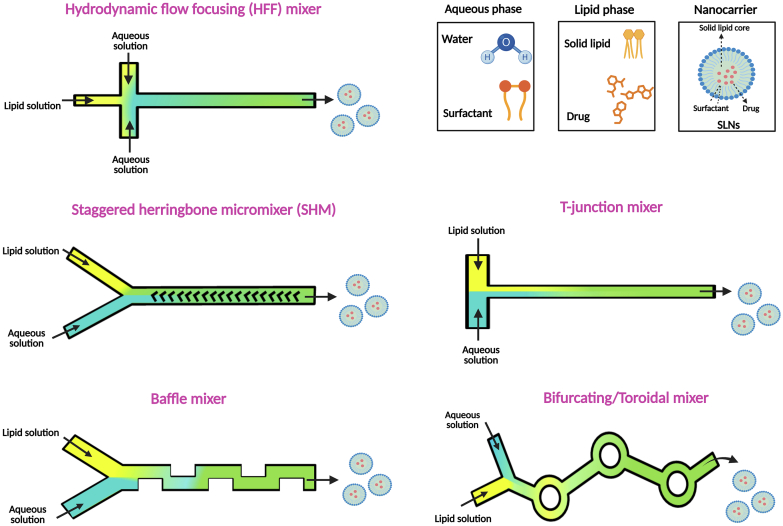
Representative microfluidic mixer designs for nanocarrier preparation. The Figure illustrates various microfluidic channel geometries commonly used to enhance mixing between lipid and aqueous phases, crucial for the controlled self-assembly of nanocarriers.

The integration of various mixing techniques into functional microfluidic mixing chip designs has significantly advanced the field, leading to the development of diverse specialized platforms specifically optimized for the preparation of lipid-based nanocarriers, including SLNs. These platforms are engineered with tailored channel architectures and flow configurations to generate precisely controlled mixing conditions, which are critically conducive to the reproducible formation and desired characteristics of nanocarriers (Nunziata et al., 2025). Fig. 6 presents several common microfluidic mixer designs employed for the efficient and controlled preparation of lipid-based nanocarriers, such as SLNs, from distinct lipid and aqueous phases. Microfluidic geometries significantly influence the encapsulation efficiency of APIs and the characteristics of the resulting nanoparticles, such as size and polydispersity. The choice of microfluidic mixer impacts how effectively different solubility profiles (hydrophilic vs. lipophilic) of APIs are encapsulated, mainly by controlling the mixing dynamics and subsequent self-assembly processes. Microfluidic platforms offer precise control over various parameters, including TFR, FRR, device design, and mixing angles, leading to better control over particle size, morphology, and encapsulation efficiency compared to traditional bulk methods (Bendre et al., 2022).

### 3.1 Hydrodynamic flow focusing mixers

Microfluidic hydrodynamic flow focusing (HFF) offers precise control over particle size, size distribution, and surface properties, surpassing the capabilities of conventional bulk methods. HFF devices typically utilize a cross-shaped geometry with three inlet streams. A central stream, often containing lipids dissolved in a water-miscible organic solvent (e.g., ethanol), is sheathed and focused by two adjacent aqueous buffer streams. The rapid and controlled mixing occurs at the stream interfaces due to diffusion and local dilution of the organic phase, which induces the precipitation and self-assembly of the lipids into nanoparticles. The narrowing of the fluid stream creates shear stress, which contributes to particle size control (Vogelaar et al., 2023; Piunti and Cimetta, 2023). HFF devices have been successfully used to encapsulate both hydrophilic and lipophilic drugs by adding them to the aqueous or organic phases, respectively. They are capable of manufacturing stealth liposomes and surface-modified liposomes, indicating versatility for various API types. For hydrophilic siRNA-loaded LNPs, HFF has been reported to achieve the best encapsulation efficiency (73 %) among tested methods, an acceptable value compared to typical ranges of 80–95 % (Maeki et al., 2022). A specific case study involved the continuous production of Vitamin *E*-loaded SLNs using a liquid flow-focusing and gas displacing method in microchannels. This method produced SLNs ranging from 50 to 280 nm in size, with a PDI of 0.264 ± 0.015 and a zeta potential of −30.6 ± 0.15 V. Key operational conditions included a lipid solution flow velocity of 0.0300 m/s, a surfactant (0.5 % Poloxamer) concentration, and a temperature of 27 °C. The advantages of this approach include its simplicity and the avoidance of high speeds, toxicological solvents, and high pressure (Yun et al., 2009). Another research conducted by Zhang et al. presented a novel approach for preparing SLNs using a microchannel system with a cross-shaped junction. The microfluidic system, consisting of a main microchannel and two branch channels, enabled the controlled interaction between a lipid solution in acetone and an aqueous surfactant solution to form SLNs. The preparation process relied on the diffusion of the lipid-solvent phase into an aqueous phase, inducing local lipid supersaturation and subsequent nanoparticle formation. The method demonstrated the potential to produce SLNs with small particle sizes, narrow size distributions, and spherical morphology under moderate experimental conditions, avoiding the extreme pressures and temperatures typical of conventional SLNs preparation techniques (Zhang et al., 2008).

### 3.2 Staggered herringbone micromixers (SHM)

The Staggered Herringbone Mixer (SHM) is an advanced passive microfluidic device designed to induce chaotic advection, a flow regime that dramatically enhances mixing efficiency beyond simple diffusion. Its design is characterized by a series of asymmetric, V-shaped grooves patterned onto the floor or ceiling of a rectangular microchannel. The orientation of these grooves is periodically alternated along the channel's length, which is the “staggered” feature of the design. As fluid flows through the channel, the asymmetric grooves generate two vertically stacked, counter-rotating vortices. This transverse flow repeatedly stretches and folds the adjacent fluid streams (e.g., the lipid-in-ethanol and aqueous phases) into increasingly thin layers. This process, known as chaotic advection, exponentially increases the interfacial area between the fluids, drastically reducing the time required for complete molecular mixing. A key operational characteristic of the SHM is that the intensity of this chaotic mixing is directly proportional to the TFR. A higher TFR results in stronger vortices, faster mixing, and consequently, the formation of smaller and more uniform nanoparticles. This provides a direct and predictable method for tuning the final particle size by simply adjusting the pump speed (Cai et al., 2023; Lin et al., 2024; Roces et al., 2020). SHMs have been successfully used to encapsulate both hydrophilic and lipophilic drugs. For example, they have been effective in formulating liposomes loaded with poorly soluble drugs like propofol, achieving significantly higher aqueous dispersions compared to their intrinsic solubility (Kastner et al., 2015). They are also for hydrophilic APIs like siRNA and mRNA and have demonstrated high encapsulation efficiency, with reports of achieving about ∼100 % encapsulation efficiencies from lab to industrial scale for LNPs. One study found that for mRNA LNPs, SHM resulted in encapsulation efficiencies of about 80 %, outperforming siRNA counterparts (Jürgens et al., 2023). In the context of SLNs and hydrophilic APIs, a recent study by Weaver et al. presented an innovative microfluidic technique for the preparation of SLNs that can encapsulate both hydrophilic and lipophilic APIs. This study utilized a Y-shaped inlet chip produced by Precision NanoSystems for the NanoAssemblr™ Benchtop, featuring etched herringbone channels. This specific device design is crucial as it, along with channel geometries, significantly impacts final formulation properties such as particle size, morphology, and encapsulation efficiency. Key parameters controlled during the microfluidic preparation process included a TFR of 4 mL/min and an FRR of 5:1 and a constant operating temperature of 60 °C to ensure all lipid components remained in solution. This study effectively prepared SLNs with particle diameters between 160 and 320 nm, utilizing trypsin as a model hydrophilic biologic and testosterone as a lipophilic control through the microfluidic approach. A formulation utilizing cetyl palmitate (CP) as the lipid core and Pluronic F68 (P68) as the surfactant exhibited excellent characteristics and achieved high encapsulation efficiencies for testosterone (90 %) and trypsin (47 %) (Weaver et al., 2024). The findings of Sommonte et al. demonstrated that the microfluidic approach presented a significant advancement in the preparation of SLNs, particularly highlighted in their development of brain-derived neurotrophic factor (BDNF)-loaded SLNs for brain delivery. The study leveraged a commercially available polycarbonate microfluidic device featuring a Y-shaped geometry and an internal staggered herringbone pattern. This design is instrumental in facilitating the nanoprecipitation process through controlled swirling and chaotic mixing of the aqueous and organic phases, enabling rapid diffusion and efficient particle formation. This microfluidic setup allows for precise control over experimental conditions, including TFR and FRR, which are critical for achieving highly homogeneous nanoparticles. By maintaining the temperature above the melting point of the lipid throughout the process, potential channel clogging was prevented. The resulting BDNF-SLNs exhibited favorable characteristics in terms of size (190.3 ± 10.1 nm) and PDI (0.180 ± 0.023), demonstrating the technique's robustness and proficiency. These findings underscore the potential of microfluidic-prepared SLNs to overcome BDNF's pharmacokinetic limitations (Sommonte et al., 2024). Another study successfully employed a microfluidic method for preparing SLNs loaded with diazoxide (DZX), demonstrating a highly controlled and reproducible production process superior to traditional techniques. The optimized microfluidic parameters, particularly the herringbone mixer design, enabled the consistent production of SLN-DZX with an average size of 180.1 ± 3.2 nm and a narrow PDI (0.125 ± 0.020), along with a high encapsulation efficiency (85.6 ± 10.2 %). The SLNs produced exhibited functional and pharmacokinetic advantages. Drug release studies revealed that DZX release was negligible in the absence of human serum but reached 97.3 % over 96 h in its presence, indicating the presence of effective intracellular release mechanisms through enzymatic degradation of the lipid matrix. These findings underscore the potential of microfluidic methods in developing SLNs with tunable physicochemical properties, efficient drug encapsulation, and controlled release profiles (Arduino et al., 2024).

A case study has unequivocally demonstrated the efficacy of microfluidics for the rapid and scalable production of SLNs using a commercially available benchtop NanoAssemblr™ instrument from Precision Nano-Systems Inc. (incorporating SHM). The system utilized two distinct inlet streams: one containing lipids dissolved in ethanol and the other an aqueous buffer. Syringe pumps were used to precisely control the flow rates and flow ratios between the two inlet streams, allowing for fine-tuning of the mixing conditions. The lipid phase comprised 1.3 mg of tristearin and 0.25 mg of mPEG-DSPE dissolved in 1 mL of ethanol (heated to 70 °C). Focusing on tristearin-based SLNs with encapsulated ovalbumin (OVA) proteins, the research meticulously characterized the influence of microfluidic process parameters on particle properties. A critical finding was the impact of the FRR on SLN size; increasing the FRR from 1:1 to 3:1 significantly reduced the mean diameter from 180 ± 65 nm to 65 ± 23 nm, with minimal further reduction at an FRR of 5:1. This size reduction, coupled with low polydispersity (0.2–0.3) and consistent negative zeta potential (−17 to −20 mV), is attributed to enhanced organic phase dilution, which mitigates lipid diffusion and particle fusion. Similarly, the TFR influenced particle characteristics, with increases from 10 mL/min to 15 mL/min or above, resulting in particle sizes of approximately 40 ± 4 nm while maintaining homogeneity. Notably, microfluidics facilitated high protein encapsulation efficiency (approximately 40 % at 0.1 mg/mL OVA), surpassing conventional methods. The observed rapid, non-zero/first-order protein release within 24 h suggests potential benefits for applications such as antigen delivery, where an initial burst is desirable. This study outlines a rapid and scalable protocol for producing SLNs, facilitating their translation from the laboratory to the clinic (Anderluzzi and Perrie, 2020).

A recent study elucidated the efficacious formulation of exenatide-loaded SLNs via a microfluidic-assisted methodology, attaining remarkable encapsulation efficiency (94.2 %) and drug loading (9.7 %), thereby marking a pivotal progression in surmounting hurdles associated with oral peptide delivery. Utilizing the NanoAssemblr system integrated with a staggered herringbone mixer, the approach harnesses the expeditious and regulated mixing characteristic of microfluidics, promoting homogeneous nanoparticle self-assembly through nanoprecipitation. Optimized process parameters, encompassing an FRR of 3:1 and a TFR of 12 mL/min, engendered SLN exhibiting a hydrodynamic diameter of approximately 120 nm, low PDI (0.13), and a positive zeta potential of +53 mV (Birro et al., 2025).

### 3.3 T and Y-junction mixers

A simple passive design where lipid-solvent and aqueous streams meet at a T- or Y-shaped junction. Mixing occurs via diffusion at the interface under laminar flow, with turbulence possible at higher Reynolds numbers (*Re* 100–4000). This approach offers notable advantages, including straightforward fabrication, rapid mixing capabilities, versatility for high-throughput production, and overall cost-effectiveness, making it an attractive option for scalable lipid nanoparticle synthesis (Abdelkarim et al., 2021; Terada et al., 2021). In a representative case study, cannabidiol (CBD)-loaded liposomes adaptable to SLNs through the incorporation of solid lipids such as soya phosphatidylcholine and cholesterol were successfully generated at a FRR of 1:3 and a TFR of 10 mL/min, yielding particles smaller than 150 nm with a PDI below 0.15 and an encapsulation efficiency of approximately 73 %; this configuration markedly improved the solubility of hydrophobic drugs, demonstrating promising potential for applications in pain management (Tiboni et al., 2021).

### 3.4 Microfluidic oscillator mixer

The microfluidic oscillator mixer offers a distinct approach that generates high-frequency oscillatory flow to achieve rapid fluid homogenization. Its mechanism relies on an elastomer diaphragm that vibrates spontaneously above a critical pumping pressure, a phenomenon that converts the otherwise laminar flow into an efficient, oscillatory mixing pattern. A primary advantage of this design is its capacity for high-throughput production, reaching up to 50 mL/min, which overcomes the low production rates that often limit other microfluidic systems (Agha et al., 2023). The performance of this mixer was investigated for the anti-solvent precipitation of SLNs using Gelucire 44/14 as the solid lipid. Across a broad lipid concentration range (10 to 300 mg/mL), the system produced SLNs with sizes varying from 50 to 240 nm and a PDI between 0.16 and 0.26. The study concluded that SLN size increased with lipid concentration and decreased with a higher anti-solvent-to-solution flow rate ratio, while the mixer's secondary chamber depth and pumping pressure were also identified as influential parameters (Xia et al., 2015).

### 3.5 Baffles and bifurcating mixers

Baffle mixers incorporate a series of perpendicular turns in fluid pathways to achieve rapid mixing of components for lipid-based nanoparticles. The iLiNP device is an example of a baffle mixer, featuring a two-dimensional design that aims to overcome issues like sample flow stagnation observed in SHMs. Kimura et al. developed the iLiNP device, which allows for fine-tuning of lipid nanoparticle size within 10 nm intervals by adjusting the flow rate, its ratio, and device dimensions. This device has demonstrated the ability to produce lipid nanoparticles with average sizes ranging from 20 nm to 100 nm (Kimura et al., 2018). While specific, detailed encapsulation efficiency values for diverse solubility profiles are not extensively provided for baffle mixers in the sources, research indicates their effectiveness for certain APIs. In a study comparing iLiNP baffle mixers with a conventional micromixer device for LNP production at varying lipid concentrations, iLiNP produced LNPs smaller than 100 nm at a POPC concentration of 10 mg/mL and sizes in the range of 130–140 nm (PDI < 0.2) when the concentration increased tenfold. The iLiNP was also found to achieve a complete mixing state within 3 ms at a TFR of 500 μL/min (FRRs of 3 and 9), suggesting its efficiency in mass production of size-controlled LNPs (Matsuura-Sawada et al., 2022). For siRNA delivery, Sato et al. also investigated the physicochemical properties of different-sized LNPs composed of a pH-sensitive cationic lipid (YSK-05), which were produced using the iLiNP device. This research showed that small-sized LNPs were better distributed in hepatocytes, though larger ones exhibited higher gene-silencing activity (Sato et al., 2016). Bifurcating mixers, sometimes referred to as toroidal mixers (e.g., NxGen™ technology), function by dividing and then recombining fluid flow multiple times. This process creates a rapidly mixed environment. While the studies primarily highlight their use for lipid-based nanomedicines like liposomes and lipid nanoparticles (LNPs), they are presented as a microfluidic platform capable of continuous manufacturing with high control over particle polydispersity and encapsulation efficiency. In a study focusing on DNA-loaded LNPs, an NxGen™ Cartridge chip equipped with a toroidal micromixer was used. It was observed that increasing the TFR up to 4 mL/min reduced the resulting LNP particle size and PDI, after which the particle size plateaued around 100 nm. Similarly, altering the FRR to 3 and above maintained particle sizes around 100 nm with low PDI values and high encapsulation efficiencies.

Table 2 provides a comprehensive overview of various microfluidic devices employed for nanocarriers. This compilation summarizes the key features of different microfluidic platforms, the types of produced nanoparticles, the operational parameters that influence nanoparticles characteristics, and the advantages and limitations associated with each approach, offering a valuable resource for researchers in the field of nanomedicine.

**Table 2:** Comprehensive overview of various microfluidic devices employed for nanocarrier production.

Type of Device	Fabrication Method	Mixing Technique	NPs Formulated	Advantages	Challenges	Applications	Ref.
HFF	Typically, soft lithography for PDMS, silicon/glass	Passive diffusive mixing due to laminar flow regime	Liposomes, PLGA NPs	Controlled particle size, narrow size distribution, enhanced properties for biomedical applications, allows for self-assembly through controlled flow, potential for high-throughput and continuous production, tunable size and surface properties, high encapsulation efficiency for polymeric NPs, allows for a variety of encapsulated cargo	Residual organic solvent, limited range of size and lamellarity, low production concentration	Drug delivery, Gene therapy, cosmeceutics, imaging agents, Tumor targeting	(Lu et al., 2016; Huang et al., 2017)
SHM	Soft lithography (for PDMS), potentially other micromachining	Chaotic advection induced by herringbone structures	Liposomes, LNPs	Improved mixing, small particles (20–150 nm), narrow PDI (<0.2), enhanced cellular uptake	Clogging, solvent swelling effects	Drug delivery, co-delivery of hydrophilic and lipophilic drugs, antitumor efficacy (e.g., curcumin-loaded liposomes)	(Amreen and Goel, 2021; Cheung and Al-Jamal, 2019)
T-junction Mixer	T-shaped chip fabricated via soft-lithography	Mixing by diffusion in laminar flow	SLNs, Liposomes, LNPs	Monodisperse droplets (coefficient of variation <5 %), high-throughput, precise size control	Requires long channels for high flow rates, solvent compatibility issues; scale-up challenge	Vaccine production (e.g., mRNA-1273), cancer drug delivery	(Cheng et al., 2024; Chen et al., 2025)
Oscillator Mixer	Piezoelectric transducers bonded to silicon, glass, PDMS, or PMMA; soft lithography; 3D printing	Oscillatory flow, acoustic streaming	SLNs (50–240 nm), polymeric PLGA (∼65 nm), Liposomes (∼80 nm)	High mixing efficiency (up to 100 %), rapid mixing, precise control over size and PDI, high throughput (up to 50 mL/min), prevents clogging	Complex fabrication and integration of transducers, potential heating issues, requires precise frequency/voltage control, typically lower flow rates	Drug delivery (e.g., budesonide in PLGA), imaging agents, therapeutic delivery, cancer therapy	(Agha et al., 2023; An Le et al., 2020)
Baffle Mixers	Soft lithography (PDMS/glass), laser cutting, 3D printing (PMMA, borosilicate glass), injection molding	Chaotic advection, Dean vortices, perpendicular turns induced by baffles	LNPs, (20–150 nm), liposomes (∼50–200 nm), PLGA (∼80 nm)	Simple design and operation, no external energy required, efficient mixing at low Reynolds numbers, high throughput potential, good for high-viscosity fluids	Prone to clogging and fouling especially with viscous solutions, limited flexibility in operational parameters, material compatibility issues (e.g., PDMS swelling with organic solvents)	Drug delivery systems (mRNA-LNPs, siRNA), gene therapy, vaccine formulation, biomedical nanoparticle production	(Yu et al., 2025; Li et al., 2025b)
Bifurcating mixers	Soft lithography, 3D printing, commercial cartridges (e.g., NxGen toroidal mixer)	Split-and-recombine (SAR) flow, chaotic advection, Dean vortexing in bifurcated paths	LNPs (30–100 nm), polymeric NPs (∼70 nm), liposomes	High reproducibility and encapsulation efficiency, scalable production (up to 20 L/h), precise control over NP size and uniformity, operates without external energy	Potential for channel blockage in complex designs, requires optimization for flow rates, solvent compatibility challenges, higher fabrication complexity for multi-stage SAR	Gene therapy (CRISPR/Cas9, siRNA delivery), catalysis, sensing applications, therapeutic nanocarrier formulation	(Seder et al., 2024; Gamage et al., 2025)

## 4 Biomedical applications of microfluidic SLNs

### 4.1 Targeted drug delivery

SLNs have certain advantages for targeted drug delivery, including enhanced bioavailability, controlled release, and reduced systemic toxicity. Such benefits are a direct outcome of their ability to encapsulate lipophilic and hydrophilic drugs and release them accurately to target tissues (Madkhali, 2022). Targeting modalities are classified under two headings: passive and active. Passive targeting takes advantage of the enhanced permeability and retention (EPR) effect regularly occurring in tumor tissues, and active targeting is done by surface modification with ligands such as antibodies or peptides to bind to specific receptors on target cells (Attia et al., 2019). Ligands are selective molecules selected to bind with highest affinity to biomarkers such as receptors or antigens overexpressed on target cells' surfaces (e.g., tumor cells or cells of the blood-brain barrier's endothelial barrier) but absent or expressed in low density on normal cells. Such selective ligand-receptor binding has several roles: it can lead to selective nanoparticle adherence to target cells on their surfaces, induce cellular internalization by mechanisms such as receptor-mediated endocytosis, and significantly increase the local concentration of the therapeutic agent at the desired site of action and thus increase efficacy while limiting systemic exposure and off-target adverse effects (Bazak et al., 2015). SLNs are primarily utilized in oncology to improve the formulation and delivery of highly potent, poorly water-soluble chemotherapeutic agents, including paclitaxel and docetaxel. Encapsulating hydrophobic drugs within the lipid core of SLNs enhances their solubility, protects them from premature degradation in the bloodstream, and modifies their pharmacokinetic profile, thereby reducing systemic toxicity and improving patient tolerance (Matsuura-Sawada et al., 2022; Sivadasan et al., 2023). The SLNs exhibit significant potential in cancer therapy, effectively tackling several obstacles linked to conventional chemotherapy. SLNs were extensively studied for breast cancer therapy. Formulations of SLNs that deliver drugs such as paclitaxel and curcumin demonstrated improved efficacy against drug-resistant breast cancer cell lines (Rahdari et al., 2025; Sarkar et al., 2025). Moreover, the active targeting of SLNs utilizing ligands like folic acid or antibodies (e.g., anti-HER2) has demonstrated an enhancement in tumor-specific accumulation and cytotoxic effects (Mo et al., 2023). SLNs provide a distinct benefit of direct pulmonary administration through inhalation. This method can attain elevated local drug concentrations at the tumor site while reducing systemic side effects (Abu Elella et al., 2024; Abdulbaqi et al., 2021). Preclinical research unraveled the prospect of SLNs to improve co-delivery of a blend of curcumin and paclitaxel to lung cancer cells and thus enhance treatment efficacy (Li et al., 2024). Microfluidic-prepared SLNs were successfully loaded with various anticancer drugs, including paclitaxel and sorafenib, and were effective against different cancer cell lines (Arduino et al., 2021b). Additionally, it has been demonstrated that iRGD-functionalized SLNs loaded with PTX (iRGD-SLN-PTX), prepared using microfluidic techniques, exhibit enhanced cytotoxicity in glioblastoma cells by binding to αvβ3 integrins, which are overexpressed in the tumor vasculature, followed by protease-triggered penetration into tumor tissues. In this study, SLNs were prepared using a co-flow geometrical setup, where the organic phase, consisting of lipid and drug, was precisely injected into the aqueous phase with a flow rate ratio of 1:5. In this system, efficient mixing was achieved to form well-characterized SLN particles with highly uniform size distribution. Microfluidics' continuous flow-based process also allowed the production of SLNs with particle sizes of 136.3 ± 3.1 nm for SLN-PTX and 137.3 ± 2.5 nm for iRGD-SLN-PTX. It had a low PDI (< 0.2), representing a unimodal distribution, and also a zeta potential of −11.2 to −36.8 mV, indicating satisfactory colloidal stability. The encapsulation efficiency of paclitaxel was also acceptable, with values of 40.4 ± 6.8 % for SLN-PTX and 31.7 ± 4.8 % for iRGD-SLN-PTX, which underscores the microfluidic system's capability to optimize drug loading during nanocarrier preparation (Arduino et al., 2021a).

**Fig. 7:**
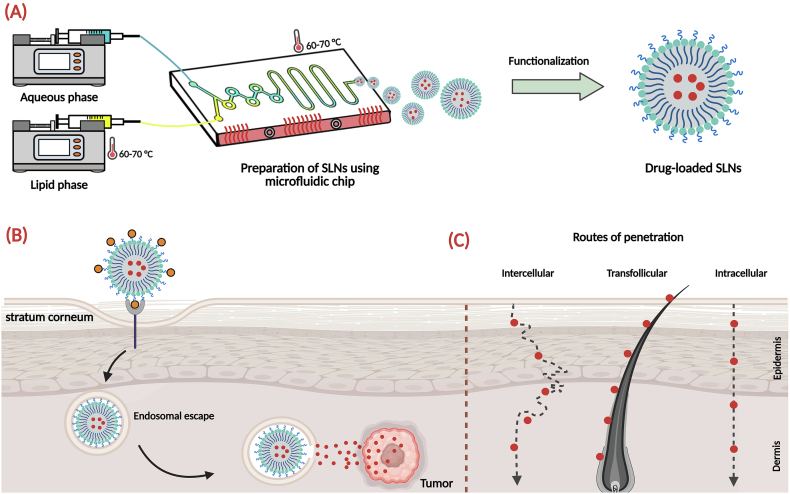
Overview of microfluidic-assisted SLN preparation and their biomedical applications, particularly for drug delivery. Depiction of the microfluidic chip system for the controlled preparation of SLNs from aqueous and lipid phases, followed by surface functionalization to create drug-loaded SLNs (A). Proposed mechanism for SLN-mediated therapeutic delivery, including interaction with the stratum corneum, subsequent cellular internalization, critical endosomal escape, and targeted drug release within tumor cells (B). Illustration of the primary pathways for SLN penetration into the skin layers, comprising intercellular, trans follicular, and intracellular routes (C).

In addition to cancer treatment, microfluidic SLNs have demonstrated effective siRNA encapsulation for cancer and retinal disease gene silencing. The narrow control over the size provided by microfluidics is also of special value when it comes to ocular drug delivery, where the corneal penetration and intraocular distribution can be affected significantly by the particle size. Tailored-in-size microfluidic SLNs can be engineered for effective and target-specific drug delivery to specific regions of the eye (Pandey et al., 2022; Sanjay et al., 2018). The delivery of drugs across the blood-brain barrier (BBB) is a significant obstacle in the management of neurological disorders, and SLNs can be utilized to address this challenge (Satapathy et al., 2021). Their small size and lipophilic characteristics may enhance translocation across the endothelial cell layer. However, passive diffusion is inefficient; consequently, surface modification is the critical factor in optimizing brain delivery. The proposed mechanisms for transport involve endocytosis by brain endothelial cells, followed by transcytosis to the brain parenchyma. A strategy that has been extensively investigated involves coating SLNs with specific surfactants, such as polysorbate 80 (Sola et al., 2023). In this regard, a study demonstrated that microfluidic SLNs loaded with diazoxide increased BBB permeability and improved therapeutic efficacy in the treatment of Friedreich's ataxia (Arduino et al., 2024). Fig. 7 provides a schematic overview of the microfluidic production and subsequent application of functionalized, drug-loaded SLNs. As depicted in panel (A), distinct lipid and aqueous phases are precisely delivered by syringe pumps into a microfluidic chip, where controlled mixing conditions facilitate the rapid and reproducible self-assembly of SLNs. Following their formation, these nanoparticles undergo functionalization, typically involving surface modification to impart specific properties or targeting capabilities, and are subsequently loaded with a therapeutic agent.

Panel (B) illustrates the encapsulation of a drug-loaded SLN from its initial interaction with a biological barrier to its subsequent cellular uptake, intracellular processing (endosomal escape), and ultimately, the targeted delivery of its therapeutic cargo to a tumor, highlighting key steps in achieving effective cancer therapy.

### 4.2 Cosmeceutical applications

The cosmetics industry is increasingly using nanotechnology to enhance the functionality and appeal of its products. When applied topically, biologically active chemicals in cosmetics, which fall in between pharmaceuticals and cosmetics, have drug-like effects. To address the drawbacks of conventional cosmetic formulations and introduce new characteristics that enhance product performance and customer satisfaction, nanotechnology is being utilized in cosmeceuticals (Kaul et al., 2018). For their uses in skin, hair, nail, and lip care, nanocosmetics and nanocosmeceuticals are being investigated in great detail (Gupta et al., 2022). Microfluidic technology is playing a transformative role in the cosmetic industry, offering numerous benefits in the formulation and production of innovative products (Hu et al., 2023). Microfluidics enables the encapsulation of novel active ingredients within nanoparticles, maximizing their efficacy upon skin contact while minimizing degradation through oxidation. This precise control over droplet properties even affects parameters such as size, formulation, and aesthetics, allowing for unparalleled adaptability over traditional batch-to-batch methods in cosmetics (Liu et al., 2021a; Park et al., 2021). The advantages of microfluidics, such as the ability to produce SLNs with a narrow size distribution and high batch-to-batch reproducibility, are inherently beneficial for the formulation of cosmetic products. In cosmetic applications, controlled particle consistency and properties are crucial because they can significantly influence factors such as skin penetration, product texture, and formulation stability. The controlled size and homogeneity achieved by microfluidic synthesis enhance the interaction of SLNs with the skin, thereby improving drug penetration (Patel et al., 2019). SLNs also offer several advantages, making them ideal for use in cosmeceutical formulations. SLNs as complex systems offer innovative solutions to overcome the powerful barrier functions of the skin. Their unique composition and nanoscale dimensions both contribute to enhanced drug permeation, increased drug stability, and precisely controlled release, rendering them invaluable for a wide range of cosmeceutical and dermatological uses (Cassayre et al., 2024). SLN formulations can utilize a sophisticated array of mechanisms to overcome the formidable barrier of the skin, thereby enhancing drug permeation and bioavailability for both topical and transdermal applications. Upon administration onto the epidermal surface, SLNs form a protective hydrophobic coating, which acts to create an occlusive layer, significantly reducing transepidermal water loss (TEWL). By cutting down water loss by evaporation, hydration of the epidermis is enhanced, particularly in the stratum corneum. Increased hydration plays a crucial role in maintaining a healthy epidermis and is hypothesized to enhance drug permeation by perturbing strongly compacted corneocytes of the stratum corneum (Santonocito and Puglia, 2025). Additionally, SLN formulations actively interact with and fluidize the lipids of the stratum corneum, enhancing drug solubilization and facilitating its diffusion throughout the cutaneous coats. The specific lipid composition of the nanocarrier system, including the carbon chain length and functional groups of the incorporated lipids, has been identified as a key mechanism promoting drug penetration (Cruz et al., 2025).

Drugs encapsulated in SLNs can permeate the skin through three routes (Fig. 7**, panel C**). The intercellular pathway, the predominant route, involves drug diffusion between the cells of the stratum corneum along a tortuous and convoluted path, which significantly increases the diffusion path length compared to the actual thickness of the stratum corneum. This route is primarily limited by the highly organized and lipophilic lipid environment, which acts as a barrier to passive diffusion. The intracellular pathway, in contrast, involves the movement of drugs across the lipid-rich regions of the corneocytes and is considered the primary route for polar drugs. The transfollicular pathway offers an alternative route through skin appendages, including hair follicles and sweat glands. Hair follicles, in particular, offer a significant pathway for larger molecules and particulate structures, bypassing the impermeable stratum corneum and serving as reservoirs for sustained drug release (Gu et al., 2022; Yu et al., 2021).

Particle size is a crucial factor in determining the efficacy of SLNs in delivering to the skin, as smaller particles enhance contact with the stratum corneum due to their increased surface area and amphiphilic surface nature. SLNs of around 100 nm can permeate deeper cutaneous levels, yet those of about 300 nm preferably localize at shallower parts of upper levels. Large SLNs, measuring approximately 1000 nm, permeate the skin poorly. For practical topical applications, optimum particle sizes for SLNs are 100–500 nm. Furthermore, the lipophilic properties of follicular ducts enhance the compatibility between lipophilic drugs and the SLN core, significantly improving drug penetration into the skin layers (Heydari et al., 2024).

Both traditional and microfluidically constructed SLNs hold much promise for topical and cutaneous drug applications by capitalizing on their ability to permeate the epidermal barrier and deliver sustained drug release to target sites. For example, SLNs are widely used in anti-aging products to deliver antioxidants, such as vitamins C and E, and retinoids, which can protect the skin from oxidative damage and increase collagen synthesis, thereby reducing the appearance of wrinkles and fine lines (Shukla et al., 2024; Alves da Silva et al., 2025). Similarly, such nanocarriers can effectively encapsulate moisturizing agents, such as hyaluronic acid and ceramides, to improve skin hydration and prevent dryness and irritation (Albakr et al., 2024). SLNs are also beneficial for the targeted delivery of UV filters, such as zinc oxide and titanium dioxide, to prevent cutaneous irritation and enhance photoprotection. Their occlusive properties improve skin hydration, and UV filters encapsulated in SLNs provide broad-spectrum photoprotection without undesirable whitening effects (Pitzanti et al., 2020; Chavda et al., 2023). Furthermore, encapsulating compounds such as coenzyme Q10 or resveratrol in microfluidic SLNs will prevent photoaging by delivering antioxidant and collagen-targeting protection (Ghasemiyeh and Mohammadi-Samani, 2020). Apart from general cutaneous applications, SLNs are targeted explicitly toward hair follicles, delivering active ingredients such as keratin and biotin to stimulate hair growth and prevent hair loss (Raszewska-Famielec and Flieger, 2022). For conditions like androgenetic alopecia, SLNs can specifically target sebaceous glands to deliver drugs like minoxidil or finasteride. By microfluidic particle size tuning, optimal follicular retention can be achieved, which increases therapeutic efficacy while minimizing systemic absorption (Tampucci et al., 2022; Roy et al., 2025).

New applications of microfluidic SLNs are continuously emerging, demonstrating their versatility in advanced therapeutic and diagnostic strategies. These applications include combining SLN formulations and microneedles to develop advanced dermal drug delivery systems to bypass hepatic drug degradation in the first phase and achieve targeted drug therapy for localized skin diseases (Gunawan et al., 2025; Kheirieh et al., 2025). Microfluidic technology is instrumental for testing and screening SLN-based drug delivery systems. The use of SLN formulations integrated with advanced organ-on-a-chip models is another promising avenue in drug modeling and screening, providing a more realistic environment for modeling advanced diseases using custom-made models, such as lung, brain, liver, and tumor-on-a-chip. Such nascent applications are a testament to the high potential of microfluidic-prepared SLNs to contribute to innovative solutions in the biomedical field (Zhang et al., 2017; Ferreira et al., 2024).

### 4.3 Comparative performance: microfluidic vs. conventional SLNs

A critical measure of success for any drug delivery platform is its performance in a biological context. While direct head-to-head in vivo studies comparing the pharmacokinetics and biodistribution of SLNs prepared by microfluidic versus bulk methods are still an emerging area of research, a growing body of evidence from in vitro and comparative formulation studies strongly suggests the superiority of the microfluidic approach. The enhanced control over nanoparticle physicochemical properties afforded by microfluidics directly translates to improved biological performance. The preparation method of SLNs, whether bulk or microfluidic, plays a pivotal role in determining SLNs' physicochemical properties, such as size, PDI, encapsulation efficiency, and surface characteristics. These attributes, in turn, influence pharmacokinetics, biodistribution, and therapeutic efficacy. Table 3 contrasts the key performance characteristics of SLNs prepared using microfluidic platforms versus conventional bulk methods.

**Table 3:** Comparative analysis of key performance indicators for SLNs prepared by microfluidic vs. conventional methods.

Performance parameter	Microfluidic-prepared SLNs	Conventionally prepared SLNs	Ref.
Particle size & PDI	Highly uniform size (often around 100–200 nm) and low PDI (typically <0.2). Example: LZ SLNs: 158.05 ± 4.86 nm, PdI 0.25 ± 0.04	Larger size, higher PDI (>0.3), broader distribution, and inadequate control over final size. Example: LZ SLNs: 180.21 ± 7.46 nm.	(Sommonte et al., 2023)
Morphology	Uniform spherical or turtle-like structures (e.g., solid cored-spherical; peculiar turtle-like surface in lysozyme SLNs via cryo-EM)	Irregular and not extremely spherical morphology, structure consists of denser lipidic concentric lamellae with a diffuse core	(Sommonte et al., 2023)
Encapsulation efficiency	Higher encapsulation efficiency due to uniform and rapid mixing, ensuring optimal drug incorporation. Examples: PTX-SLN: 54 ± 3 % SFN-SLN: 79 ± 1 % Cur-SLNs: over 60 %	Often low and variable, example: PTX-SLN: 42 ± 1.5 % SFN-SLN: 65 ± 2.4 %. Cur-SLNs: ∼40 %.	(Arduino et al., 2021b; Kim et al., 2025)
Drug release profile	Tunable and sustained release profiles, often with an initial burst effect followed by prolonged release, and release can be triggered by enzymes (e.g., lipase). Examples: PTX-SLNs: 10 % in 6 h (without lipase), 100 % in 72 h (with lipase), BDNF-SLNs: 20 % in the first 4 h (without lipase), steady within 72 h, DZX-SLNs: 97.3 % released within 96 h in the presence of human serum, OVA-loaded SLNs: up to 90 % released within the first 24 h (rapid release)	Conventional prepared SLNs showed no drug release for PTX and SFN in 120 h when tested without human serum	(Arduino et al., 2021a; Sommonte et al., 2024; Arduino et al., 2024; Anderluzzi and Perrie, 2020)
Cytotoxicity & Biocompatibility	High biocompatibility; empty SLNs show >80 % cell viability at high concentrations and are non-hemolytic (<5 % at appropriate dilutions), Exenatide-loaded SLNs maintained over 85 % cell viability up to 20 μg/mL of exenatide.	Methods like microemulsion require high surfactant levels, raising potential toxicity concerns	(Birro et al., 2025; Weaver et al., 2024)
Cellular uptake	Uniform particles enable effective surface functionalization for targeted uptake (e.g., with iRGD peptide), SLNs (especially with sizes between 120 and 200 nm) are able to bypass the reticuloendothelial system (RES) absorption, extending circulation time and improving delivery to target sites	Lower due to size	(Arduino et al., 2021a)
Pharmacokinetics & Biodistribution	More consistent due to high uniformity and excellent colloidal stability in plasma	More variable due to polydispersity and potential for aggregation in biological fluids	(Arduino et al., 2021b)
Therapeutic efficacy	Demonstrates enhanced cytotoxicity against cancer cells compared to free drug and shows superior anti-inflammatory effects in neuroinflammation models. Examples: iRGD-functionalized SLNs demonstrated enhanced anti-cancer activity of paclitaxel by increasing cytotoxicity and providing more efficient tumor targeting and penetration in both 2D and 3D tumor models, BDNF-SLNs showed an enhanced decrease in nitrite production and NOS mRNA levels in an in vitro neuroinflammatory model (TBI-like model) compared to plain BDNF, DZX-SLNs led to a significantly greater improvement in cell viability and a discernible reduction in mitochondrial ROS production in frataxin-deficient fibroblasts	Efficacy can be compromised by lower drug loading, poor stability, and uncontrolled release	(Arduino et al., 2021a; Sommonte et al., 2024; Arduino et al., 2024)

## 5 Integration of AI/ML and developing complex multi-functional formulations

Artificial intelligence (AI) and machine learning (ML) are increasingly applied to solve difficult problems of scientific and engineering disciplines (He et al., 2023; Puttegowda and Ballupete Nagaraju, 2025). ML algorithms can automatically gain knowledge from data sets, discern underlying patterns, and optimize their performance with no human help, paving the way for applications such as chemical processing optimization, prediction of material properties, rapid discovery, and increased efficiencies. The growth in popularity of open-source ML software has prompted including these computational tools as part of experimental workflow and data assessment pipelines, creating a trend toward data-driven strategies (Lu, 2023; Tan, 2025). Such a paradigm enables rapid and comprehensive exploration of large data sets, discerning subtle interplays and optimizing multi-aspect processes, which otherwise become difficult to handle experimentally (Aal E Ali et al., 2024; Long et al., 2025). Large and complex experimental data often surpass human analytical capability, making AI/ML unavoidable for derivation of insight and promulgating innovation within a broad array of domains (Gupta et al., 2023; Xu et al., 2021).

The prediction performance of ML models in nanocarrier formulation is highly dependent on the dataset size and diversity. For predicting complicated quality variables like particle size or encapsulation efficiency, robust generalization requires datasets spanning from hundreds to thousands of entries; for instance, a model trained on 6398 lipid nanoparticle formulations obtained over 90 % prediction accuracy. While smaller datasets (∼100–200 points) may be sufficient for basic calculations, their reliability generally needs augmentation via techniques like virtual screening. Furthermore, real-time process monitoring applications demand much bigger datasets (>1000–5000 points) to capture temporal process variability efficiently (Öztürk et al., 2018; Li et al., 2025a). Integration of AI/ML with microfluidic devices has transformative potential in terms of optimizing synthesis of nanoparticles (Shirzad et al., 2025; Alavinejad et al., 2025). These algorithms automate device design, reduce experimental workflow, interpret large data sets, and predict optimal synthesis parameters. AI/ML-based approaches optimize geometries of microchannels, fluidic characteristics of fluidic flows, and mixing performance. Artificial neural networks (ANNs), for instance, have been utilized to predict particle sizes within microfluidic devices and design chips with a pre-determined droplet size in droplet-based microfluidics (Chen and Lv, 2022; Saravanakumar and Cicek, 2023). AI/ML also permits fine-tuning of parameters with high specificity, such as flow rates, reagent concentrations, temperature, and mixing ratios, to produce nanoparticles with wanted physicochemical properties, including size, morphology, and drug entrapment performance (Bai and Zhang, 2025; Wang et al., 2022). AI/ML strategies optimize nanocarrier formulation by predicting how lipid constitution and ratios of excipients influence characteristics such as drug encapsulation efficiency, targeting, and stability. ML models assess the impacts of particle size and surface charge on nanocarrier performance, enabling the design of enhanced therapeutic formulations (Haghighi et al., 2024; Jena et al., 2024; Chintan et al., 2024). AI-driven optimization pipelines in pharmaceutical manufacturing, including SLN production, use machine learning models to optimize parameters such as lipid ratios, flow rates, and quality attributes. To make sure that AI-driven optimization pipelines work for GMP-compliant manufacturing, you need to use a lot of different methods, starting with strict model validation. This includes functional testing, calibration, and statistical performance evaluation using metrics such as sensitivity and area under the curve (AUC). All of these depend on high-quality, preprocessed data. Standardization is subsequently achieved by implementing a QbD framework. This validated quality by design (QbD) framework is finally embedded within Standard Operating Procedures (SOPs) to ensure batch-to-batch consistency, facilitate regulatory compliance, and address the inherent challenges of scaling reproducible microfluidic processes from the laboratory to industrial production (Niazi, 2025). Multiple reports have documented the successful applications of AI/ML during the preparation of microfluidic nanocarriers (Alam, 2023; Habeeb et al., 2024). AI/ML has been utilized in optimizing lipid composition as well as LNPs' flow parameters, improving size control and RNA encapsulation efficacy of siRNA and mRNA delivery. Similarly, AI has optimized lipid concentrations, solvent ratios, and flow settings during the preparation of SLNs, thereby enhancing both drug-loading and drug-release profiles (Seo et al., 2023; Sato et al., 2024; Walsh et al., 2014). ML pipelines predict as well as optimize important SLNs attributes, such as hydrodynamic diameters, polydispersity index, and encapsulation efficacy, based on microfluidic input parameters, including total flow rates, aqueous-to-organic phase ratios, and reagent concentrations. Considering the lipid-based nature shared by liposomes and SLNs, ANN models trained on liposome size and dispersity prediction can often be adaptable for SLNs optimization (Egorov et al., 2021; Eugster et al., 2024; Dorsey et al., 2024). A key case study applied support vector machines (SVMs) and feed-forward ANNs to guide microfluidic preparation of liposomes containing curcumin. Researchers prepared over 200 formulations by systematically changing their flow rates, lipid concentrations, and volumetric mixing ratios using a benchtop microfluidic setup. The obtained models performed well in predicting liposome size, dispersity, and stability, enabling them to identify suitable optimum formulation parameters that meet the desired design criteria. This study is an excellent example of how ML can enable the rapid development of lipid-structured nanocarriers, including SLNs, for nanomedicine applications. (Di Francesco et al., 2023). Similarly, Suriyaamporn et al. demonstrated that AI/ML outperforms traditional methods, such as the design of experiments (DoE), enabling the precise and efficient formulation of SLNs with tailored properties (Suriyaamporn et al., 2024). The integration of AI into formulation processes advances nanomedicine and enhances the development of targeted drug delivery systems.

## 6 Considerations for industrial translation and scalability

The development of various products based on nanoformulations is increasing globally; however, a bottleneck for their commercialization is maintaining their properties during large-scale production at an industrial scale. To this end, microfluidic-based solutions have garnered the attention of the pharmaceutical and biotechnology industries. In this way, Pfizer-BioNTech could produce mRNA-loaded LNPs as a COVID-19 vaccine through the parallelization of microfluidic impingement jet mixers in a continuous manner at a rate of 100 million doses per month. Although details of this production have not been disclosed, it is expected that at this scale, the rate of flow injections into each mixer may increase up to hundreds of milliliters or even a few liters per minute, using high-pressure positive displacement pumps, to suffice the production demand. At such flow rates, the flow speed through the entire system, particularly the mixing chip, is very high. An employed micromixing chip for such applications should generate very low pressure loss while maintaining high mixing efficiency. Additionally, the chip geometry should not have any dead zones that could lead to clogging. Regarding the production of SLNs on an industrial scale, the primary concern is maintaining a uniform elevated temperature throughout the entire production line to prevent phase change or solidification of the lipid phase. This inhibits any fouling or clogging due to the phase change of solid components in a formulation and guarantees uniform and homogeneous nano-assembly of SLNs. In this way, heat loss from the flow paths of formulation solutions, such as tubing and connectors, should be minimized through insulation, or warming up the flow paths should be implemented if necessary (Zhang et al., 2023).

Inter-batch consistency is typically achieved by maintaining operational and compositional conditions throughout the production process. Unlike conventional methods, in which nanoassembly of nanoparticles occurs in bioreactors and the operational conditions change over time, the physicochemical properties in microfluidic mixers remain constant over time, as nanoassembly of particles occurs continuously at steady-state conditions. To ensure inter-batch consistency, the operational conditions of each microfluidic unit, including the flow rates of solutions or the pressure of each pump, as well as temperature should be monitored. For a specific formulation, operational conditions such as flow rates should be maintained fixed during production time. Notably, positive-displacement pumps should be employed for the injection of solutions into microfluidic units to avoid flow variations during the injection process (Huanbutta et al., 2024). Additionally, in-line measurement of particle size for quality control purposes could be applied for continuous production of nanocarriers. This allows for rapid modification of operational conditions or stopping the production process right after production of nanocarriers in the mixing chip to avoid inter-batch inconsistencies. The most advanced strategy, as discussed in our review, involves integrating AI and ML with in-line process analytical technologies (PAT). The real-time data from PAT sensors can be fed into an AI control loop. This system can identify if a particular channel or batch is deviating from the validated state and could be designed to make autonomous adjustments to system-wide parameters (e.g., pump pressure) or flag specific units for maintenance. This approach moves from simple quality control to active, real-time quality assurance, which is the ultimate goal for robust, large-scale manufacturing (Gerzon et al., 2022).

Controlling the risk of cross-contamination and the feasibility of cleaning microfluidic chips are also very critical considerations for industrial manufacturing compared to research applications.

Cleaning and washing the microfluidic chip after each use is quite effective in eliminating the risk of contamination. In general, the same solvent used in the formulation is also used for cleaning purposes. Importantly, in cGMP conditions required by biotech and pharma companies, all wetted paths should be washed with KOH or NaOH and sterile water (Water for Injection, WFI) at predefined intervals. Additionally, after completing the cleaning procedure, all wetted paths and tubes are filled with ethanol to maintain their sterility for subsequent use. Additionally, the deployment of sterile and single-use chips is another option to prevent cross-contamination in research settings. In cGMP conditions, the use of sterile, ready-to-use tubing assemblies is also common for specific applications (Pattanayak et al., 2021).

## 7 Conclusion and future perspectives

Microfluidics has become a revolutionary platform for SLNs preparation, a considerable leap forward from conventional bulk preparation. It provides unprecedented control over key physicochemical characteristics, routinely delivering nanoparticles with reduced mean diameters, reduced polydispersity, and significantly improved drug encapsulation efficacies for small molecules and delicate biologics. Automated, continuous microfluidic processing is a significant benefit, which delivers high batch-to-batch reproducibility necessary for industrial scale-up and clinical translation, in turn overcoming an essential drawback of standard methods. For industrial scale-up, maintaining temperature at elevated levels through the fluidic path is required since formulation solutions for SLNs production are generally warmed up. Importantly, all wetted materials and fluidic paths including pumps, mixing chip, tubing, connectors, etc. should meet requirements of current good manufacturing practice (cGMP) conditions.

In the future, microfluidic technology development would be spurred on through advancements in several key fields. The integration of AI and ML holds immense promise for developing autonomous, self-correcting synthesis systems, though this is contingent on the availability of large, high-quality training datasets. A concerted effort is also required to navigate regulatory pathways by establishing standardized, GMP-compliant protocols integrated with in-line PAT. Microfluidics is ideal for PAT because the small scale and continuous nature of the process make it easy to integrate sensors directly into the microchannels. Finally, the precision of microfluidics should be leveraged to formulate next-generation complex SLNs. The demonstrated success in encapsulating a diverse range of APIs from small molecules to biologics paves the way for engineering sophisticated nanocarriers for multidrug co-delivery or for protecting highly sensitive gene-based therapies. Addressing these challenges and pursuing these future directions will be crucial for unlocking the full potential of microfluidic-prepared SLNs, solidifying their role in the next generation of advanced nanomedicines.

## Availability of data and materials

Available data are presented in the manuscript.

## CRediT authorship contribution statement

**Mohammad Javad Javid-Naderi:** Writing – review & editing, Writing – original draft, Methodology, Formal analysis, Conceptualization. **Seyed Ali Mousavi Shaegh:** Writing – review & editing, Writing – original draft, Supervision, Project administration, Conceptualization.

## Funding

SAMS would like to acknowledge financial support from the Stem Cell Initiative under grant number 51526 to conduct this research. Also, technical comments from the Clinical Research Development Unit of Ghaem Hospital are greatly appreciated.

## Declaration of competing interest

The authors declare that they have no known competing financial interests or personal relationships that could have appeared to influence the work reported in this paper.

## Data Availability

Data will be made available on request.
